# Relative Abundance of SARS-CoV-2 Entry Genes in the Enterocytes of the Lower Gastrointestinal Tract

**DOI:** 10.3390/genes11060645

**Published:** 2020-06-11

**Authors:** Jaewon J. Lee, Scott Kopetz, Eduardo Vilar, John Paul Shen, Ken Chen, Anirban Maitra

**Affiliations:** 1 Sheikh Ahmed Center for Pancreatic Cancer Research and the Department of Translational Molecular Pathology, The University of Texas MD Anderson Cancer Center, Houston, TX 77030, USA; jjlee3@mdanderson.org; 2 Department of Surgical Oncology, The University of Texas MD Anderson Cancer Center, Houston, TX 77030, USA; 3 Department of Gastrointestinal Medical Oncology, The University of Texas MD Anderson Cancer Center, Houston, TX 77030, USA; skopetz@mdanderson.org (S.K.); evilar@mdanderson.org (E.V.); jshen8@mdanderson.org (J.P.S.); 4 Department of Clinical Cancer Prevention, The University of Texas MD Anderson Cancer Center, Houston, TX 77030, USA; 5 Department of Bioinformatics and Computational Biology, The University of Texas MD Anderson Cancer Center, Houston, TX 77030, USA; kchen3@mdanderson.org

**Keywords:** SARS-CoV-2, COVID-19, gastrointestinal tract, scRNA-seq

## Abstract

There is increasing evidence of gastrointestinal (GI) infection by severe acute respiratory syndrome coronavirus 2 (SARS-CoV-2). We surveyed the co-expression of SARS-CoV-2 entry genes *ACE2* and *TMPRSS2* throughout the GI tract to assess potential sites of infection. Publicly available and in-house single-cell RNA-sequencing datasets from the GI tract were queried. Enterocytes from the small intestine and colonocytes showed the highest proportions of cells co-expressing *ACE2* and *TMPRSS2*. Therefore, the lower GI tract represents the most likely site of SARS-CoV-2 entry leading to GI infection.

## 1. Introduction

COVID-19, the disease caused by severe acute respiratory syndrome coronavirus (SARS-CoV-2), has rapidly spread throughout the world and was declared a pandemic by the World Health Organization, thus leading to a rapid surge in the efforts to understand the mechanisms of transmission, methods of prevention, and potential therapies. While COVID-19 frequently manifests as a respiratory infection [1], there is evidence for infection of the gastrointestinal (GI) tract [1,2,3,4,5] with documented viral RNA shedding in the stool of infected patients [2,4,6]. In this study, we aimed to investigate the co-expression of *ACE2* (encoding angiotensin converting enzyme 2) and *TMPRSS2* (encoding transmembrane serine protease 2), the products of which are required for SARS-CoV-2 entry into mammalian cells [7], from single-cell RNA sequencing (scRNA-seq) datasets of five different parts of the GI tract: esophagus, stomach, pancreas, small intestine, and colon/rectum. We also queried the co-expression of *TMPRSS4*, which promotes viral entry into human intestinal epithelial cells in combination with TMPRSS2 [8]. We found predominant co-expression of *ACE2* and *TMPRSS2* in the enterocytes of the lower GI tract, with progenitor and stem-like epithelial cells demonstrating highest proportions of *ACE2*, *TMPRSS2*, and *TMPRSS4* co-expression, especially in the small intestine, which suggests a potential mechanism for GI manifestations of acute COVID-19 infection. 

## 2. Materials and Methods 

### 2.1. Public Data Acquisition

Publicly available single-cell sequencing datasets from esophagus [9,10], stomach [11], pancreatic islets [12,13,14,15], small intestine [16,17], and colon/rectum [18,19] were obtained as described in Table 1. 

### 2.2. Single-Cell Dissociation of Normal Colon Samples

For in-house normal colon samples, a total of seven patients were recruited at the University of Texas MD Anderson Cancer Center through written informed consent following Institutional Review Board approval (protocols LAB10-0982 and PA12-0327). Single cell dissociation was performed following a standard protocol: tissues were minced with sterile surgical scalpels to approximately 1 mm fragments and resuspended in 0.5 mg/ml Liberase TH (Sigma-Aldrich, St. Louis, MO, USA) followed by incubation at 37 °C for 15 min with constant agitation. Liberase was quenched with equal volume of 1% bovine serum albumin (BSA) from (Thermo Fisher, Waltham, MA, USA) and cells were resuspended in Accutase (Sigma-Aldrich) followed by incubation at 37 °C for 15 min with constant agitation. Dissociated cells were passed through a 40 μm strainer and resuspended in 0.04% BSA for subsequent viability analysis and counting. Relevant cell data for the colorectal dataset, including the expression of marker genes and SARS-CoV-2 entry genes, are provided in Data S1.

### 2.3. Single-Cell RNA Sequencing Library Generation, Sequencing, and Alignment

Single-cell RNA sequencing (scRNA-seq) library generation and sequencing were performed using the 3’ Library Construction Kit (10x Genomics, Pleasanton, CA, USA) following the manufacturer’s recommendations. Single cell data processing was performed using standard Cell Ranger RNA pipeline (10x Genomics) using hg19 as a reference.

### 2.4. Downstream Single-Cell RNA Analysis

All downstream single-cell analyses were performed in R v3.6.2 (https://www.r-project.org/) using Seurat v3.1.0 [20], and analysis codes are provided in Data S2. Preprocessing included removal of genes expressed in less than three cells, removal of cells containing less than 200 genes, and removing cells with high percentage of mitochondrial genes based on distribution with general cutoff ranging between 20% and 50%. Given the size of the datasets, integration was performed using reciprocal principal component analysis as a part of standard Seurat workflow (https://satijalab.org/seurat/v3.0/integration.html). After initial integration of datasets for each organ, epithelial cells were subsetted for further analysis. Seurat normalized count greater than zero was defined as presence of expression. Gene Ontology (GO) enrichment analysis was done on geneontology.org [21,22] using genes that were positively correlated with *ACE2* in all small intestine and colorectal cells with Pearson’s *r* > 0.1, which were obtained using *cor()* function.

## 3. Results

**Figure 1:**
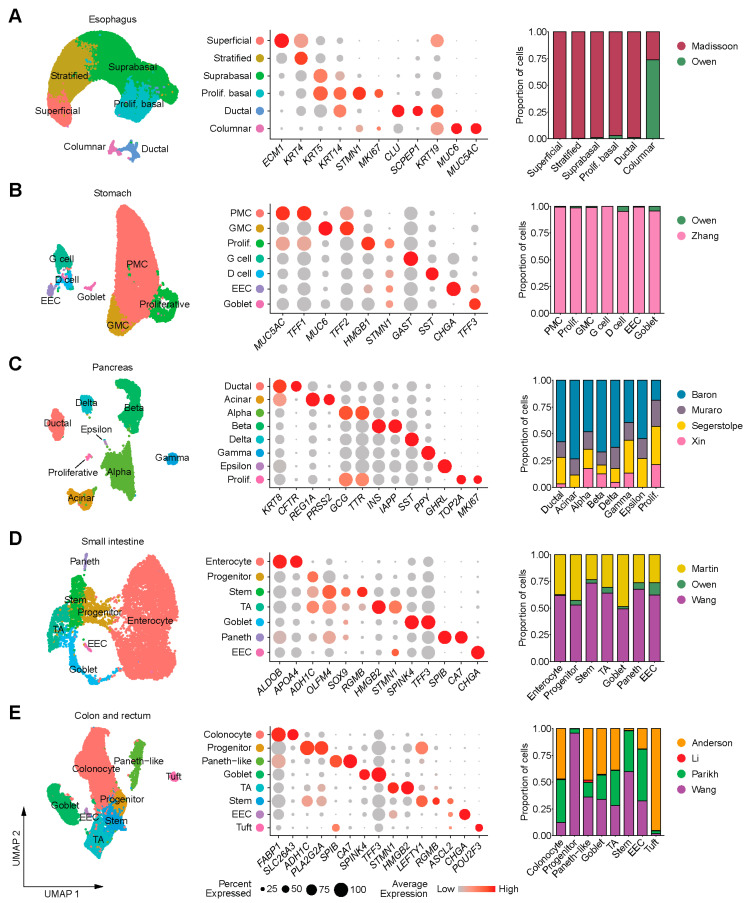
Uniform manifold approximation and projection (UMAP) plots (left) of single-cell RNA-seq (scRNA-seq) data from (**A**) esophagus, (**B**) stomach, (**C**) pancreas, (**D**) small intestine and colon and (**E**) rectum; bubble plots showing expression levels of cell-type marker genes (middle) and bar plots depicting the distribution of different datasets in each cell type (right). EEC: Enteroendocrine cell, GMC: Antral basal gland mucous cell, PMC: Pit mucous cell, Prolif: Proliferative, TA: Transit-amplifying cell.

Analysis of scRNA-seq was performed separately for each gastrointestinal (GI) segment and available cell identities from the original studies were assigned to the new clusters after confirming the expression of relevant marker genes (Figure 1). Overall, there were 82,626 cells from the esophagus, 21,399 cells from the stomach, 13,407 cells from the pancreas, 9059 cells from the small intestine, and 24,898 cells from the colon and rectum (Table 1). Datasets in each organ contained most, if not all, cell types identified, although some datasets such as Owen [10] and Li [19] were much smaller compared to others (Figure 1, Table 1). The proportions of cells expressing ACE2 were approximately 1*3*-fold lower in the upper GI tract (esophagus, stomach, duodenum; 1.04% or 1084/104,174 cells) than in the lower GI tract (ileum, colon, rectum; 14.06% or 4754/33,808 cells), and overall, higher proportions of cells expressed *TMPRSS2* than *ACE2* throughout the GI tract (Figure S1A,B). The percentages of cell types co-expressing *TMPRSS2* and *ACE2* in individual datasets are provided in Table S1.

**Figure 2:**
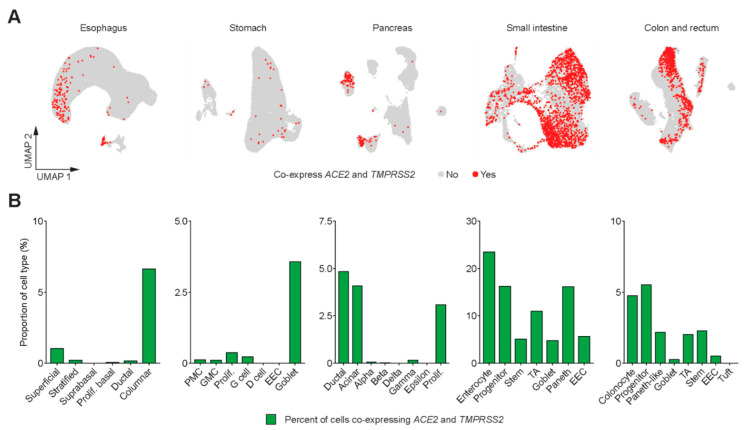
SARS-CoV-2 entry gene co-expression in cells of the gastrointestinal (GI) tract. (**A**) UMAP plots depicting cells that co-express *ACE2* and *TMPRSS2*. (**B**) Bar plots depicting proportions of cell types in the GI tract that co-express *ACE2* and *TMPRSS2*.

Of note, higher proportions of esophageal columnar cells—which were mostly from Barrett’s esophagus samples—co-expressed ACE2 and TMPRSS2 compared to the native squamous epithelium (Figure 2). Pancreatic ductal and acinar cells co-expressed ACE2 and TMPRSS2 but endocrine cells did not show detectable co-expression (Figure 2). Parenthetically, the expression of TMPRSS2 in acinar cells (Figure S1B) underscores the rationale for using a TMPRSS2 inhibitor (camostat mesylate) in acute pancreatitis. In fact, this agent is now undergoing early phase clinical trials in COVID-19 patients [7].

**Figure 3:**
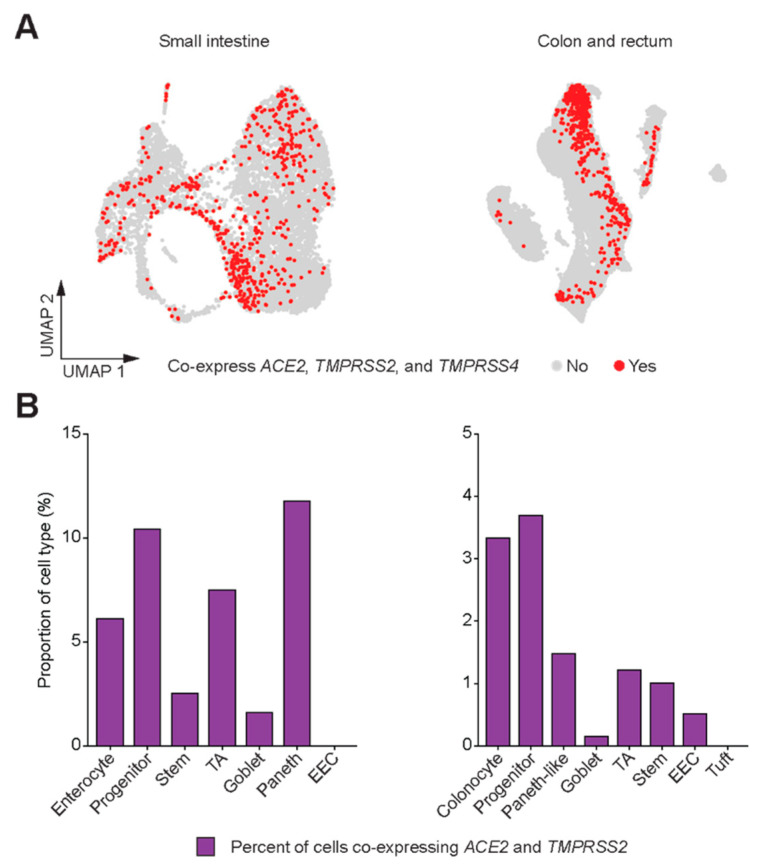
Co-expression of *ACE2* and *TMPRSS2* with *TMPRSS4*. (**A**) UMAP plots depicting cells that co-express *ACE2*, *TMPRSS2*, and *TMPRSS4* in small intestine and colon/rectum. (**B**) Bar plots depicting proportions of cell types in the small intestine and colon/rectum that co-express *ACE2*, *TMPRSS2*, and *TMPRSS4*.

Taken together, within the GI tract, the co-expression of ACE2 and TMPRSS2 transcripts was highest in the small intestine and colon/rectum (Figure 2A). Greater than 20% of enterocytes from the small intestine and approximately 5% of colonocytes co-expressed ACE2 and TMPRSS2 (Figure 2B). Recent evidence suggests that another serine protease TMPRSS4 also promotes SARS-CoV-2 entry into human enterocytes and has an additive effect with TMPRSS2 [8]. Therefore, we looked for the expression of TMPRSS4 throughout the GI tract as well as its co-expression with ACE2 and TMPRSS2 in small intestine and colon/rectum (Figure 3A, Figure S1C). While approximately 5% of enterocytes and 3% of colonocytes co-expressed all three genes, progenitor cells in the small intestine (approximately 10%) and colon/rectum (approximately 3.5%) as well as Paneth cells in the small intestine (approximately 12%) demonstrated higher proportion of cells co-expressing all three genes (Figure 3B).

**Figure 4:**
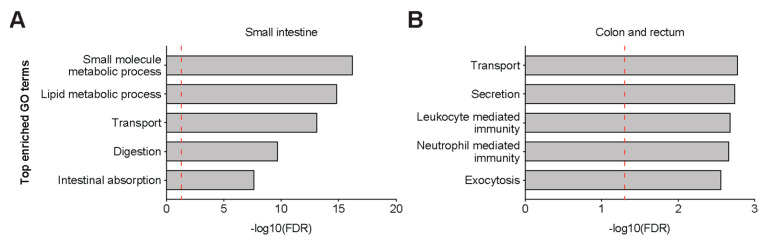
*ACE2* co-regulatory networks in the lower gastrointestinal (GI) tract. Bar plots of top Gene Ontology (GO) biological process terms enriched in 321 genes that are positively correlated with *ACE2* expression in (**A**) the small intestine and in (**B**) the colon/rectum. Vertical dashed line (red) represents significance threshold at false discovery rate (FDR) of 0.05.

Analysis of 321 genes that were positively correlated (Pearson’s *r* > 0.1) with ACE2 in the small intestine (Table S2) revealed the enrichment of functional Gene Ontology (GO) terms such as metabolic, digestion, and transport pathways (Figure 4A), thus confirming the inherent digestive and absorptive functions of enterocytes. Interestingly, 135 genes positively correlated with ACE2 in the colon and rectum (Table S3) showed enrichment of not only secretion-associated pathways but also immune-related processes (Figure 4B), which may be upregulated in functional colonocytes due to the microbiome.

## 4. Discussion

Current evidence supports the GI tract as a site of COVID-19 infection with up to 50% patients reporting digestive symptoms [5]. Our results confirm the co-expression of SARS-CoV-2 entry genes *ACE2* and *TMPRSS2* in a subset of epithelial cells in the GI tract, especially functional enterocytes from the lower GI tract, and provide potential insights into why entero-colitic symptoms may arise in acute COVID-19 infection. 

A recent report by the Human Cell Atlas (HCA) Lung Biological Network surveyed the co-expression of *ACE2* and *TMPRSS2* in multiple organ systems in the body and showed the co-expression of SARS-CoV-2 entry genes in enterocytes and progenitor cells of the ileum and colon but focused their analysis on the cells of the respiratory tract [23]. Our study includes additional previously unpublished data from the colon and confirms the co-expression of *ACE2* and *TMPRSS2* in the lower GI tract is more prevalent than the upper GI tract. The entry of SARS-CoV-2 into the host cell begins by the binding of viral spike glycoprotein with ACE2 protein followed by processing of the spike glycoprotein by TMPRSS2 leading to membrane fusion, and recent evidence suggests an additive effect of TMPRSS4 in viral entry [8]. Therefore, we also surveyed the co-expression of *TMPRSS4* with other viral entry genes in the lower GI tract and found that the progenitor cells had the highest co-expression of all three genes suggesting a mechanism for prolonged GI infection.

Another study also exploring the expression of *ACE2* and *TMPRSS2* from scRNA-seq data of the GI and respiratory tract demonstrated higher mean expression of these two genes in the lower GI tract than the respiratory tract and confirmed ACE2 protein expression in the mucosal cells of the lower GI tract [24]. While our study lacks protein expression data of *ACE2* and *TMPRSS2*, other functional studies utilizing small intestine enteroids showed evidence of active infection of intestinal epithelial cells by SARS-CoV-2 [8,25], confirming the possibility of COVID-19 infection in the GI tract. Interestingly, one of the studies demonstrated the inactivation of SARS-CoV-2 by simulated human GI secretions [8], suggesting that while the GI tract is susceptible to infection, it may not be a route of further transmission.

Our results parallel the findings from recent studies that revealed the co-expression of *ACE2* and *TMPRSS2* in olfactory epithelium [26] and intrahepatic cholangiocytes [27], which may explain atypical COVID-19 symptoms and laboratory findings such as anosmia/dysgeusia and transaminitis. Our results further support the feasibility of SARS-CoV-2 entry into the GI tract, especially in the small intestine, with implications for GI infection.

## Figures and Tables

**Table 1:** Summary of datasets used in the study.

Tissue	Dataset	Number of Cells	Sequencing Depth (Total Counts *)	Source	Notes
Esophagus	Madissoon [9]	81,380	7.58 × 10^8^	Seurat object from HCA	
	Owen [10]	1246	1.81 × 10^8^	Counts matrix from Supplementary Data	Contains Barrett’s esophagus samples
	All	82,626			
Stomach	Owen [10]	221	1.75 × 10^7^	Counts matrix from Supplementary Data	
	Zhang [11]	21,178	7.11 × 10^7^	Counts matrix (GSE134520)	Included non-atrophic gastritis and chronic atrophic gastritis
	All	21,399			
Pancreatic islets	Baron [12]	7744	4.61 × 10^7^	Single Cell Experiment objects from Hemberg’s Group	All samples were enriched for endocrine cells
	Muraro [13]	2002	n/a		
	Segerstolpe [14]	2121	1.03 × 10^9^		
	Xin [15]	1540	n/a		
	All	13,407			
Small intestine	Martin [16]	3333	1.37 × 10^7^	Counts matrices (GSE134809)	Excluded diseased samples (Crohn’s)
	Owen [10]	149	1.61 × 10^7^	Counts matrix from Supplementary Data	
	Wang [17]	5577	5.97 × 10^7^	Counts matrix (GSE125970)	
	All	9059			
Colon and rectum	Parikh [18]	7665	1.65 × 10^7^	Counts matrices (GSE116222)	Excluded diseased samples (ulcerative colitis)
	Wang [17]	7610	1.30 × 10^8^	Counts matrix (GSE125970)	
	MDACC	9429	4.92 × 10^7^	In-house data	
	Li [19]	194	1.65 × 10^8^	Counts matrix (GSE81861)	
	All	24,898			

* Unique molecular identifier counts from filtered Seurat object were summed to obtain total counts with the exception for Muraro and Xin datasets, which only contain normalized and log-transformed counts. HCA: Human Cell Atlas, MDACC: MD Anderson Cancer Center.

## Supplementary Materials

The following are available online at https://www.mdpi.com/2073-4425/11/6/645/s1, Figure S1: Proportions of *ACE2*-, *TMPRSS2*-, and *TMPRSS4*-expressing cells in GI tract; Table S1: Summary of percent of cell types co-expressing *ACE2* and *TMPRSS2* reported by individual datasets; Table S2: List of 321 genes positively correlated with *ACE2* in the small intestine; Table S3: List of 135 genes positively correlated with *ACE2* in the colon and rectum; Data S1: Cell meta data, dimension coordinates, and expression of marker genes in colon/rectal data set. Data S2: Analysis code.
