# Supplementary material for: Relative Abundance of SARS-CoV-2 Entry Genes in the Enterocytes of the Lower Gastrointestinal Tract
Source: Genes (Basel). 2020 Jun 11;11(6):645. doi: 10.3390/genes11060645 (PMC7349178; doi:10.3390/genes11060645)
Supplement: Supplementary file 1 [file genes-11-00645-s001.zip › Figure S1.docx]

**Supplementary Information**


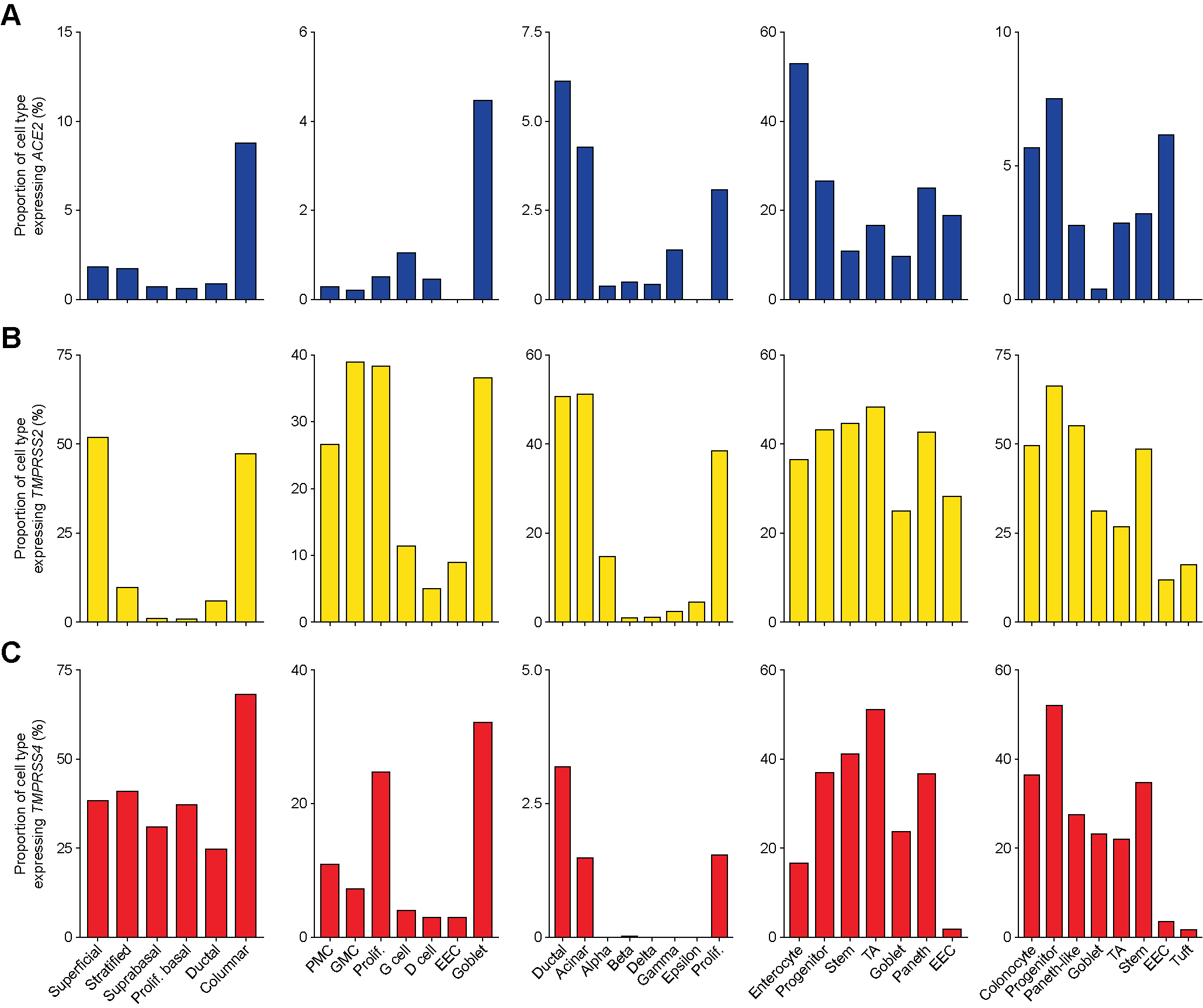


**Supplementary Figure 1.** Expression of SARS-CoV-2 entry genes throughout gastrointestinal (GI) tract. Bar plots representing proportions of cell types in the GI tract that express (**A**) *ACE2,* (**B**) *TMPRSS2* and (**C**) *TMPRSS4.* Each bar represents a single percent value. EEC: enteroendocrine cell; GMC: antral basal gland mucous cell; PMC: pit mucous cell; Prolif: proliferative; TA: transit-amplifying cell.

Supplementary Table Legends

Supplementary Table 1. Summary of percent of each cell type co-expressing *ACE2* and *TMPRSS2* reported by individual datasets.

Supplementary Table 2. List of 321 genes positively correlated with *ACE2* in small intestine (Pearson’s *r* > 0.1).

Supplementary Table 3. List of 135 genes positively correlated with *ACE2* in the colon and rectum (Pearson’s *r* > 0.1).
